# Substance use patterns, sociodemographics, and health profiles of harm reduction service recipients in Burlington, Vermont

**DOI:** 10.1186/s12954-024-00995-y

**Published:** 2024-04-05

**Authors:** Tyler G. Erath, Rosalie LaCroix, Erin O’Keefe, Stephen T. Higgins, Richard A. Rawson

**Affiliations:** 1 Vermont Center on Behavior and Health, Burlington, VT USA; 2 Department of Psychiatry, University of Vermont, University Health Center, 1 S. Prospect St., Burlington, VT 05401 USA; 3 Howard Center Safe Recovery, Burlington, VT USA; 4 Center on Rural Addictions, University of Vermont, Burlington, VT USA

**Keywords:** Harm reduction, Syringe service program, Drug use, Stimulants, Opioids, Fentanyl, Overdose, MOUD, Substance use treatment

## Abstract

**Background:**

Understanding current substance use practices is critical to reduce and prevent overdose deaths among individuals at increased risk including persons who use and inject drugs. Because individuals participating in harm reduction and syringe service programs are actively using drugs and vary in treatment participation, information on their current drug use and preferred drugs provides a unique window into the drug use ecology of communities that can inform future intervention services and treatment provision.

**Methods:**

Between March and June 2023, 150 participants in a harm reduction program in Burlington, Vermont completed a survey examining sociodemographics; treatment and medication for opioid use disorder (MOUD) status; substance use; injection information; overdose information; and mental health, medical, and health information. Descriptive analyses assessed overall findings. Comparisons between primary drug subgroups (stimulants, opioids, stimulants-opioids) of past-three-month drug use and treatment participation were analyzed using chi-square and Fisher’s exact test.

**Results:**

Most participants reported being unhoused or unstable housing (80.7%) and unemployed (64.0%) or on disability (21.3%). The drug with the greatest proportion of participants reporting past three-month use was crack cocaine (83.3%). Fentanyl use was reported by 69.3% of participants and xylazine by 38.0% of participants. High rates of stimulant use were reported across all participants independent of whether stimulants were a participant’s primary drug. Fentanyl, heroin, and xylazine use was less common in the stimulants subgroup compared to opioid-containing subgroups (*p* < .001). Current- and past-year MOUD treatment was reported by 58.0% and 77.3% of participants. Emergency rooms were the most common past-year medical treatment location (48.7%; *M* = 2.72 visits).

**Conclusions:**

Findings indicate high rates of polysubstance use and the underrecognized effects of stimulant use among people who use drugs—including its notable and increasing role in drug-overdose deaths. Crack cocaine was the most used stimulant, a geographical difference from much of the US where methamphetamine is most common. With the increasing prevalence of fentanyl-adulterated stimulants and differences in opioid use observed between subgroups, these findings highlight the importance and necessity of harm reduction interventions (e.g., drug checking services, fentanyl test strips) and effective treatment for individuals using stimulants alongside MOUD treatment.

**Supplementary Information:**

The online version contains supplementary material available at 10.1186/s12954-024-00995-y.

## Background

In 2021, an estimated 106,699 drug-involved overdose deaths occurred in the United States (US), a record-high and astonishing twofold increase from 52,404 deaths in 2015 [1, 2]. Much of the current overdose epidemic is driven by a rise in synthetic opioids such as illicitly manufactured fentanyl, a contributor to approximately 71,000 deaths in 2021 [2, 3]. However, psychomotor stimulants including cocaine and methamphetamine are also contributing to record numbers of unintentional poisonings and overdose deaths, both alone and in combination with fentanyl, dramatically rising from approximately 12,000 deaths in 2015 to over 53,000 deaths in 2021 [2–4].

These recent data indicate a “fourth wave” of the overdose crisis wherein co-occurring use of fentanyl and stimulants is a primary contributor to overdose deaths [5, 6]. Indeed, underpinning this public health crisis are differences in the most used polysubstance combinations, which vary by geographic location and rurality. For example, whereas national data indicate that co-use of methamphetamine and fentanyl is the most common drug combination found in overdose deaths in much of the US, cocaine and fentanyl is the most common combination in the northeastern US [5, 7].

The provision of harm reduction services is a primary component of addressing the ongoing overdose epidemic, as indicated by its inclusion in the Biden-Harris Administration’s approach to substance use and the U.S. Department of Health and Human Services Overdose Prevention Strategy [8]. Syringe service programs (SSPs) are community-based programs that provide a range of harm reduction services [9]. Since their initial creation to help reduce and prevent the transmission of blood-borne infectious diseases (e.g., human immunodeficiency virus [HIV], hepatitis C virus [HCV]), SSPs have expanded to offer many harm reduction services and evidence-based interventions now commonly alongside providing sterile syringes for people who use drugs (PWUD). These additional services often include overdose education and naloxone distribution, case management and counseling, adulterant test strips (e.g., fentanyl, xylazine), safer smoking supplies, and referrals and linkage to substance use treatment, medical care, and mental health treatment among others [10–14]. The provision of medication for opioid use disorder (MOUD) is another emerging service with 32% of SSPs from the National SSP Evaluation Survey (*n* = 158 SSPs) offering on-site treatment in 2021 [15].

Amidst an everchanging drug landscape, understanding of current substance use practices is important to reduce and prevent overdose deaths—both in general and among individuals who are at an increased risk for overdose and other drug-related harms, such as persons who inject drugs (PWID) in particular [16, 17]. SSPs are an ideal setting to inquire about current substance use practices, as their “low barrier” approach is well-liked, offering non-judgmental services and supports for individuals who often face stigma in other healthcare settings [16, 18]. Because most individuals who receive services at SSPs are actively using drugs and vary in treatment and recovery service participation, collecting information on their current drug use provides a unique window into the drug use ecology of communities. The goal of this observational study was to inform future intervention services and treatment provision by assessing drug use practices, sociodemographics, and health profiles of individuals participating in a low-barrier, community-based SSP in Burlington, Vermont that also provides a range of other harm reduction services and on-site MOUD treatment. A primary focus was to examine how one’s primary (i.e., preferred) drug affected drug-use practices, treatment participation, and other related findings.

## Methods

### Study participants and setting

The study population consisted of 150 individuals participating in harm reduction services in Burlington, Vermont. Howard Center Safe Recovery is primarily a SSP that provides sterile syringe services and other harm reduction services including free fentanyl and xylazine test strips, Narcan overdose reversal kits, and safer smoking supplies. Other treatment and service programs are also available on-site including low-barrier buprenorphine access, case management, HIV and HCV counseling, drug treatment counseling, and free legal clinics, among others. Safe Recovery is an anonymous program, thus, no identifiable information is collected or stored about participants.

### Procedures

All study procedures were conducted on-site at the SSP. This study was approved by the University of Vermont Institutional Review Board. All data were collected between March and June 2023 by a trained SSP staff member. Individuals participating in services were informed by a SSP staff member about an opportunity to participate in the study. If interested, prospective participants were provided with an information sheet. To protect anonymity, verbal informed consent was obtained from each participant and a consent process documentation form was completed. Individuals who consented to participate in the study were asked to answer a series of questions, described in detail below; all procedures were conducted in a private room. Completion of this study was one-time and accomplished in one visit. To be eligible, participants had to meet the following criteria: (a) be 18 years or older, (b) report past 30-day drug use, and (c) participation in harm reduction services at Safe Recovery. For their time and participation, participants were compensated with a $25 gift card.

### Materials

The primary data collection instrument was the University of Washington Alcohol and Drug Abuse Institute’s Washington State SSP Health Survey [19–21]. Areas queried on this structured survey included questions on (a) sociodemographics, housing, and employment status; (b) treatment and MOUD status; (c) substance use; (d) injection information; (e) overdose information; and (f) mental health, medical care, and other health information.

Sociodemographic questions asked about age, race/ethnicity, gender, monthly legal income, and being in jail or prison in the last year. Housing questions included current living situation and housing status. Current employment status was recorded. MOUD status was assessed via questions on current and past-year treatment.

Regarding drug use, participants reported which drug(s) was their primary (i.e., preferred) drug via an open-ended question. Additionally, participants were asked to indicate which drugs they had used in the past three months from the following list: (1) heroin, (2) methamphetamine, (3) methamphetamine and heroin mixed together (i.e., goofball), (4) crack cocaine, (5) powder cocaine, (6) cocaine and heroin mixed together (i.e., speedball), (7) fentanyl (and was it purposeful use?), (8) xylazine, and (9) alcohol. For each drug used, participants reported the route(s) of administration (injected, smoked, snorted) and the number of days used in the past seven days. If the drug was injected, participants also reported the number of days injected in the past week. Percentages for the use of each drug were calculated as the proportion of participants endorsing past-three month use among all participants and the sample size for each subgroup. Percentages for each route of administration were calculated as the proportion of participants endorsing route use among those who reported use of the drug. The average number of days used and injected in the past week was calculated across participants who reported use of the drug.

For participants reporting injection, a series of follow-up questions included the number of people they were picking up syringes for, number of days they picked up syringes from SSP in the past month, age of first injection, number of injections on an average day, number of times a syringe is used before discarded, frequency of being alone when injecting, frequency of injecting in a public place, and any abscesses or skin infections, blood clots, or endocarditis.

Overdose information included the number of past year personal overdoses, past year number of overdoses observed, and use of naloxone/Narcan kit in the past three months. Two mental health questions assessed a participant’s concern for depression and anxiety. Regarding medical information, participants reported their type of health insurance, time of last HIV and HCV test, if they ever had HCV, and if ever treated for HCV. For medical care, participants reported treatment places where services had been received within the past year.

### Analysis

Descriptive statistics and frequency distributions were generated to assess sociodemographics, injection and overdose information, and mental health, health and service utilization. Comparisons between primary drug subgroups of past-three-month use of each drug and treatment participation were analyzed using chi-square and Fisher’s exact test. All analyses were performed using GraphPad Prism 10 (Boston, MA).

## Results

Results for all tables and topics are reported for all participants (*n* = 150), followed by columns for the subgroups categorized by primary drug. Three subgroups were created based on participant reported primary drug(s); the subgroups include stimulants (*n* = 72), opioids (*n* = 56), and both stimulants and opioids (*n* = 17; hereafter referred to as stimulants-opioids). Five participants who reported alcohol as their primary drug are included in the overall but not the subgroup analyses.

### Sociodemographics, housing, employment, and incarceration status

Table 1 contains detailed information on sociodemographics, housing, employment, and incarceration status. The mean age across all participants was approximately 39 years old; 51.3% were male and most were white (90.0%), aligning with state demographics. Most participants were either unhoused (46.0%) or in temporary or unstable housing (34.7%), with 18.7% of participants reporting permanent housing. The most common living situations were alone (38.7%) or with a significant other (34%). For employment, 64.0% of participants were unemployed, 21.3% on disability, and 4.7% indicated full-time work. Forty participants (26.7%) reported being in jail or prison in the last year.

**Table 1:** Sociodemographics, housing, employment, and incarceration status

Variable	All participants (n = 150)	
	*N*	%
Mean age	38.91	
Legal Monthly Income	$524	
Gender		
Male	77	51.3
Female	71	47.3
Transgender	1	0.7
Other	1	0.7
Race		
White	135	90.0
Latinx/Hispanic	4	2.7
Black	4	2.7
American Indian/Alaska Native	5	3.3
Other	7	4.7
Housing status		
Unhoused	69	46.0
Temporary/unstable	52	34.7
Permanent	28	18.7
Not reported	1	0.7
Living situation		
Alone	58	38.7
With significant other	51	34.0
With friends/parents	27	18.0
Other	14	9.3
Employment status		
Unemployed	96	64.0
Disability	32	21.3
Full-time work	7	4.7
Other	15	10.0
Jail or prison in last 12 months		
Yes	40	26.7
No	110	73.3

Table 2 contains information on current and past year treatment for all participants and each of the three subgroups with results organized by type of treatment. Regarding MOUD, 87 participants (58.0%) reported current treatment, with 33.3% and 24.7% of participants receiving methadone or buprenorphine/suboxone, respectively. Past-year MOUD treatment was reported by 116 participants (77.3%). There was a statistically significant difference in current buprenorphine/suboxone treatment between the primary drug subgroups (*p* = 0.016).

**Table 2:** Current and past year treatment

Variable	All participants (n = 150)		Stimulants main drug (n = 72)		Opioids main drug (n = 56)		Stimulants and opioids main drug (n = 17)		*p* value
	*N*	%	*N*	%	*N*	%	*N*	%	
*Current treatment*									
None	52	34.7	20	27.8	23	41.1	7	41.2	.263
Methadone	50	33.3	24	33.3	20	35.7	6	35.3	.957
Buprenorphine/Suboxone	37	24.7	24	33.3	9	16.1	1	5.9	.016*
Outpatient	23	15.3	14	19.4	7	12.5	2	11.8	.589
12-step/recovery group	1	0.7	1	1.4	0	0.0	0	0.0	.999
*Past year treatment*									
None	32	21.3	14	19.4	11	19.6	5	29.4	.640
Methadone	66	44.0	26	36.1	32	57.1	8	47.1	.059
Buprenorphine/Suboxone	50	33.3	29	40.3	13	23.2	5	29.4	.119
Outpatient	24	16.0	16	22.2	7	12.5	1	5.9	.192
Inpatient	15	10.0	6	8.3	6	10.7	3	17.6	.502
12-step/recovery group	2	1.3	2	2.8	0	0.0	0	0.0	.614

*p < .05

### Substance use

Table 3 contains detailed information on drug use for all participants and each subgroup with results organized by drug. The term *Used* refers to reported use in the past three months.

**Table 3:** Substance use

Substance	All participants (n = 150)		Stimulants main drug (n = 72)		Opioids main drug (n = 56)		Stimulants and opioids main drug (n = 17)		*p* value
	*N*	%	*N*	%	*N*	%	*N*	%	
Crack Cocaine									
* Used*	125	83.3	61	84.7	43	76.8	17	100.0	.072
Injected	10	8.0	6	9.8	3	7.0	0	0.0	
Smoked	122	97.6	61	100.0	41	95.3	16	94.1	
Snorted	3	2.4	3	4.9	0	0.0	0	0.0	
Not used	25	16.7	11	15.3	13	23.2	0	0.0	
Days used past week	3.54		3.58		3.10		4.88		
Days injected past week	2.44		4.00		.67		0		
Powder Cocaine									
* Used*	49	32.7	21	29.2	18	32.1	8	47.1	.366
Injected	30	61.2	8	38.1	14	77.8	6	75.0	
Smoked	10	20.4	4	19.0	4	22.2	2	25.0	
Snorted	19	38.8	13	61.9	5	27.8	1	12.5	
Not used	101	67.3	51	70.8	38	67.9	9	52.9	
Days used past week	1.35		1.4		.77		2.71		
Days injected past week	1.71		2.50		1.00		2.33		
Methamphetamine									
* Used*	78	52.0	37	51.4	26	46.4	13	76.5	.092
Injected	47	60.3	17	45.9	19	73.1	9	69.2	
Smoked	44	56.4	22	59.5	17	65.4	5	38.5	
Snorted	21	26.9	11	29.7	7	26.9	3	23.1	
Not used	72	48.0	35	48.6	30	53.6	4	23.5	
Days used past week	3.56		4.08		3.27		3.00		
Days injected past week	3.75		5.44		2.89		2.78		
Heroin									
* Used*	101	67.3	33	45.8	51	91.1	16	94.1	< .001*
Injected	72	71.3	20	60.6	39	76.5	12	75.0	
Smoked	36	35.6	12	36.4	18	35.3	5	31.3	
Snorted	21	20.8	9	27.3	7	13.7	5	31.3	
Not used	49	32.7	39	54.2	5	8.9	1	5.9	
Days used past week	5.05		3.53		5.71		6.33		
Days injected past week	5.01		3.80		5.51		5.83		
Fentanyl (by itself or mixed)									
* Used*	104	69.3	37	51.4	50	89.3	16	94.1	< .001*
Injected	67	64.4	18	48.6	37	74.0	11	68.8	
Smoked	38	36.5	17	45.9	18	36.0	3	18.8	
Snorted	16	15.4	8	21.6	5	10.0	3	18.8	
Not used	46	30.7	35	48.6	6	10.7	1	5.9	
* Purposeful use?*									
Yes	43	41.3	7	18.9	28	56.0	8	50.0	.002*
Days used past week	4.93		3.29		5.55		6.18		
Days injected past week	5.03		4.46		5.16		5.80		
Speedball (cocaine and heroin mixed)									
* Used*	21	14.0	5	6.9	9	16.1	6	35.3	.008*
Injected	15	71.4	2	40.0	8	88.9	4	66.7	
Smoked	9	42.9	3	60.0	5	55.6	1	16.7	
Snorted	3	14.3	1	20.0	2	22.2	0	0.0	
Not used	129	86.0	67	93.1	48	85.7	11	64.7	
# Days used past week	2.24		3.00		2.25		2.20		
# Days injected past week	2.14		4		1.57		2.75		
Goofball (meth and heroin mixed)									
* Used*	33	22.0	8	11.1	16	28.6	9	52.9	< .001*
Injected	28	84.8	7	87.5	14	87.5	7	77.8	
Smoked	9	27.3	3	37.5	4	25.0	2	22.2	
Snorted	3	9.1	1	12.5	1	6.3	1	11.1	
Not used	117	78.0	64	88.9	40	71.4	7	41.2	
# Days used past week	3.13		3.75		2.88		3.00		
# Days injected past week	3.14		4.29		2.64		3.00		
Xylazine									
* Used*	57	38.0	14	19.4	31	55.4	12	70.6	< .001*
Injected	34	59.6	6	42.9	19	61.3	9	75.0	
Smoked	16	28.1	4	28.6	9	29.0	3	25.0	
Snorted	5	8.8	2	14.3	1	3.2	2	16.7	
Not used	93	62.0	58	80.6	25	44.6	5	29.4	
Days used past week	4.83		3.33		5.32		4.80		
Days injected past week	5.47		5.67		5.45		5.33		
Alcohol									
* Used*	67	44.7	34	47.2	17	30.4	11	64.7	.024*
Not used	83	55.3	38	52.8	39	69.6	6	35.3	
Days used past week	2.80		2.50		2.29		2.75		

*Used* = use in the past 3 months

*p < .05

#### Stimulants main drug subgroup

Seventy-two participants identified stimulants as their primary drug. Crack cocaine was the primary drug for 45 participants (62.5%), methamphetamine for 19 participants (26.4%), powder cocaine for 6 participants (8.3%), and crack cocaine and powder cocaine for 2 participants (2.8%). Crack cocaine was the stimulant and drug overall with the highest proportion of participants reporting use in the past three months (*n* = 61; 84.7%). Smoking was the most endorsed route of administration (100%) although approximately 10% of participants also reported injecting. Use of methamphetamine and powder cocaine in the past three months was reported by 37 participants (51.4%) and 21 participants (29.2%). Cumulatively, 46 participants (63.9%) reported use of opioids in the past three months, with 33 participants (45.8%) reporting fentanyl use and 37 participants (51.4%) reporting heroin use.

#### Opioids main drug subgroup

Fifty-six participants identified opioids as their primary drug. Heroin was the primary drug for 42 participants (75%), fentanyl for 7 participants (12.5%), heroin and fentanyl for 3 participants (5.4%), heroin, fentanyl, and xylazine for 3 participants (5.4%) and methadone for 1 participant (1.8%). The drugs with the highest proportion of participants reporting use in the past three months were heroin (*n* = 51; 91.1%) and fentanyl (*n* = 50; 89.3%). The most endorsed route of administration was injection for heroin (76.5%) and fentanyl (74.0%). Cumulatively, 49 participants (87.5%) reported use of stimulants in the past three months, with 43 participants (76.8%), 26 participants (46.4%), and 18 participants (32.1%) reporting use of crack cocaine, methamphetamine, and powder cocaine, respectively.

#### Stimulants and opioids main drug subgroup

Seventeen participants identified both stimulants and opioids as their primary drug. Crack cocaine and heroin were the primary drugs for 12 participants (70.6%), methamphetamine and heroin for 2 participants (11.8%), and powder cocaine and fentanyl for 1 participant (5.9%); 2 participants (11.8%) reported preference for any available drug. Crack cocaine, heroin and fentanyl were the drugs with the highest proportion reporting use in the past three months with use reported by 17 participants (100.0%), 16 participants (94.1%), and 16 participants (94.1%). The most endorsed route of administration was injection for heroin (75.0%) and fentanyl (68.8%) and smoking for crack cocaine (94.1%).

#### Subgroup differences

Differences in drugs used, frequency of use, and routes of administration were observed across the subgroups. Regarding opioid use, there was a statistically significant difference between the subgroups for past-three-month use of fentanyl (*p* < 0.001) and heroin (*p* < 0.001), as well as xylazine (*p* < 0.001). Use of fentanyl was more prevalent in the stimulants-opioids (94.1%) and opioids (89.3%) subgroups compared to the stimulants subgroup (51.4%). Purposeful use of fentanyl was different between subgroups (*p* = 0.002) with greater proportional use in the opioids (56.0%) and stimulants-opioids (50.0%) subgroups than the stimulants subgroup (18.9%). Injection was the most common route of administration among users in the opioids (74.0%), stimulants-opioids (68.8%), and stimulants subgroup (48.6%) although smoking was almost equally endorsed by users in the stimulants subgroup (45.9%). Use and subgroup differences for heroin were similar to fentanyl. Heroin use was proportionally much higher in the stimulants-opioids (94.1%) and opioids (91.1%) subgroups than the stimulants subgroup (45.8%) and injection the most common route of administration. Xylazine use was greater in the stimulants-opioids (70.6%) and opioids (55.4%) subgroups than the stimulants subgroup (19.4%).

Regarding stimulant use, there was no significant difference between the subgroups for past-three-month use of any stimulant. Crack cocaine use was proportionally highest in the stimulants-opioids subgroup (100.0%), followed by the stimulants (84.7%) and opioids (76.8%) subgroups. Methamphetamine use was highest for the stimulants-opioids subgroup (76.5%) and comparatively lower in the stimulants (51.4%) and opioids (46.4%) subgroups. Whereas users in the stimulants subgroup were most likely to smoke methamphetamine (59.5%), users in the opioids and stimulants-opioids subgroups were most likely to inject (73.1% and 69.2%). However, those in the stimulants subgroup who did inject did so more frequently (i.e., 5.4 days in the past week) than participants in the opioid-containing subgroups. Powder cocaine use was proportionally highest for the stimulants-opioids subgroup (47.1%) followed by the opioids (32.1%) and stimulants (29.2%) subgroups. Like methamphetamine, injection was the most common route of administration among users in the opioids (77.8%) and stimulants-opioids subgroups (75.0%) compared to snorting in the stimulants subgroup (61.9%).

### Substance use among participants in current MOUD treatment

Examination of drug use among the 87 participants (58.0%) in current MOUD treatment was undertaken to better understand this subpopulation of the overall sample (see Additional file 1: Table S1). High rates of stimulant use were reported by participants currently on MOUD. The stimulant used by the most participants in the past three months was crack cocaine (*n* = 77; 88.5%) followed by methamphetamine (*n* = 36; 41.4%) and powder cocaine (*n* = 31; 35.6%). For both these latter drugs, injection was the most common route of administration. Opioid use was also prevalent among participants on MOUD. Fentanyl had the highest proportional use in the past three months (*n* = 60; 69.0%) and injection the most common route of administration (60.0%). Among other drugs, 29 participants (33.3%) reported use of xylazine with injection the most endorsed route of administration (62.1%).

### Injection information

Ninety participants (60.0%) reported injecting a drug (Table 4). The average age of first injection of any drug was 23 years old. The average number of past-week days injected and injections per day was 5.34 and 5.43, respectively. Most participants reported either never injecting alone (42.2%) or only sometimes alone (30.0%). The percentage reporting any abscesses or skin infections was 43.3%. Blood clots or blood infections and endocarditis were reported by 12.2% and 5.6%. Skin infections were more commonly reported in the opioids (56.8%) and stimulants-opioids (41.7%) subgroups than the stimulants (28.1%) subgroup.

**Table 4:** Injection information

Variable	All participants (n = 90)		Stimulants main drug (n = 32)		Opioids main drug (n = 44)		Stimulants and opioids main drug (n = 12)	
	*M*	Range	*M*	Range	*M*	Range	*M*	Range
# Other people getting syringes for	0.85	0–10	.88	0–10	0.95	0–5	0.58	0–2
# Days syringes picked up in past month	4.70	0–30	4.88	0–30	4.77	0–30	4.58	0–15
Age of first injection of any drug	22.95	9–46	24.61	9–46	21.40	13–41	22.50	15–33
Years since initiating injecting	14.81	0–49	16.19	0–49	15.23	0–43	13.73	7–21
# Days injected in past week	5.34	0–7	4.66	0–7	5.71	0–7	6.33	2–7
# Injections per day	5.43	1–20	4.21	1–10	5.49	1–20	7.82	3–20
# Times syringe used before discard	1.85	1–27	2.21	1–27	1.57	1–6	2.25	1–10

	*N*	%	*N*	%	*N*	%	*N*	%
*Frequency of being alone when injecting*								
Never	38	42.2	11	34.4	18	40.9	8	66.7
Sometimes	27	30.0	10	31.3	14	31.8	3	25.0
Most times	19	21.1	6	18.8	11	25.0	1	8.3
Always	6	6.7	5	15.6	1	2.3	0	0
*Frequency of injecting in public*	89				43			
Never	34	38.2	17	53.1	12	27.9	4	33.3
Sometimes	34	38.2	10	31.3	19	44.2	5	41.7
Most times	14	15.7	2	6.3	8	18.6	3	25.0
Always	7	7.9	3	9.4	4	9.3	0	0
*Injection-related health*								
Abscess or skin infection	39	43.3	9	28.1	25	56.8	5	41.7
Blood clot or blood infection	11	12.2	4	12.5	5	11.4	2	16.7
Endocarditis	5	5.6	1	3.1	4	9.1	0	0

### Overdose information

Table 5 contains detailed information on overdoses. The average number of personally experienced overdoses in the past year was 0.7, with the stimulants subgroup reporting the fewest personal overdoses among the subgroups (*M* = 0.32). The average number of overdoses a participant witnessed in the past year was 3.3. 113 participants (76.9%) reported having naloxone or a Narcan kit on them in the past three months.

**Table 5:** Overdose information

Variable	All participants (n = 147)		Stimulants main drug (n = 72)		Opioids main drug (n = 54)		Stimulants and opioids main drug (n = 16)	
	*M*	Range	*M*	Range	*M*	Range	*M*	Range
# Overdoses in past year	0.73	0–10	0.32	0–3	1.11	0–10	1.36	0–8
# Overdoses seen in past year	3.26	0–20	2.42	0–14	4.49	0–20	3.15	0–10

	*N*	%	*N*	%	*N*	%	*N*	%
Naloxone/Narcan kit on person in last three months	113	76.9	51	70.8	42	77.8	16	100

### Mental health, health, and medical information

Table 6 contains detailed mental health, health, and medical information. Concern about depression and anxiety was high among all participants. Approximately 81% and 88% of participants were concerned about depression and anxiety, respectively. Most participants reported an HIV and HCV test within the past year (64.4% and 60.4%). Medicaid was the primary type of health insurance (93.3%). Regarding medical care, the most common place participants reported past-year treatment was from the emergency room (ER)/urgent care (48.7%) with an average of 2.7 visits per person. The next most used medical care services were the doctor’s office (30.7%), SSP (5.3%), and jail/prison (5.3%).

**Table 6:** Mental health, health, and medical information

Variable	All Participants (n = 150)	
	*N*	%
*Concern about depression*	148	
Very	51	34.5
Somewhat	69	46.6
Not sure	28	18.9
*Concern about anxiety*	148	
Very	76	51.4
Somewhat	54	36.5
Not sure	18	12.2
*Time since last HIV test*	149	
Within past year	96	64.4
Over year ago	48	32.2
Never tested	5	3.4
*Time since last Hepatitis C test*	147	
Within past year	90	60.4
Over year ago	48	32.2
Never tested	9	6.0
*Ever had Hepatitis C*	142	
Yes	80	56.3
No	62	43.7
*Ever treated for Hepatitis C*		
Yes	39	26.0
No	111	74.0
*Type of health insurance*	149	
Medicaid	139	93.3
Medicare	3	2.0
Private	2	1.4
None	4	2.7
Other	1	0.7
*Past year medical care*		
Emergency Room	73	48.7
*Avg. # visits*	*2.72*	
Hospital admission	1	0.7
Doctor’s office/tribal clinic	46	30.7
Mobile medical van	0	0.0
Syringe exchange program	8	5.3
Jail/prison	8	5.3
Other	6	4.0
Didn’t need/get care	43	28.7

## Discussion

In this observational study, sociodemographics, drug use practices, treatment participation, and health profiles of individuals participating in harm reduction services in Burlington, Vermont were explored overall and by primary drug subgroups. High rates of stimulant use were reported by all participants whether stimulants, opioids, or both were identified as their “primary drug”. Considering the notable and growing prevalence of stimulants in overdose deaths [4, 5], these findings highlight a critical need for evidence-based interventions for stimulant use (e.g., Contingency Management) [7, 22] including providing such interventions in SSPs and other harm reduction settings for individuals who may be interested.

These findings illustrate an illicit drug supply that now more than ever includes both stimulants and fentanyl [23, 24]. We sought to better understand nuance of drug use practices and other related outcomes by categorizing participants by their primary (i.e., preferred) drug resulting in three subgroups—users of stimulants, opioids, and a combination of stimulants and opioids. Crack cocaine was the drug with the greatest proportion of individuals reporting use in the past three months across all participants, aligning with epidemiological data indicating cocaine as the most used stimulant in the Northeast US [5, 6]. However, subgroup evaluations revealed crack cocaine was the drug with the highest proportion reporting use for the stimulants and stimulants-opioids subgroups only. In the opioids subgroup, fentanyl and heroin had the highest proportion reporting use. These results suggest that most service recipients whose primary drug is an opioid also regularly used stimulants. Comparatively, service recipients who primarily use stimulants used opioids to a lesser extent. Examining drug use by routes of administration also provided insight into subgroup differences. For example, injection of methamphetamine and powder cocaine was the most endorsed route for the opioid subgroups. However, injection was less common in the stimulant subgroup for these drugs, with smoking methamphetamine and snorting powder cocaine preferred. Such findings have implications for drug-involved overdoses and other health-related harms [25, 26].

Approximately 70% of participants reported using fentanyl; however, purposeful use was much lower (41.3%). Purposeful fentanyl use was significantly higher in the opioid subgroups than the stimulant subgroup. The increasing prevalence of fentanyl-adulterated stimulants may be exacerbating overdoses if used unknowingly or inadvertently by individuals who report that they primarily use stimulants [27, 28]. As such, this finding highlights the critical importance and necessity of harm reduction interventions such as drug checking services [29], naloxone distribution [10], and fentanyl test strips [12] (the latter two services are offered at this SSP) for people who primarily use stimulants as well as those who primarily use opioids. Commonly, participants whose primary drug was an opioid reported assuming fentanyl was in their drug supply and referred to heroin and fentanyl interchangeably.

Current participation in MOUD treatment was reported by 58.0% of participants. When asked about MOUD treatment in the past year, this number increased to 77.3%. These data suggest that among this sample of harm reduction service recipients, many individuals on MOUD continue to use illicit drugs in general and stimulants in particular. Thus, these data suggest that additional services may be needed to help service recipients reduce or stop their drug use.

Collecting information from individuals participating in harm reduction programs provides insight into the drug use ecology that is not currently being captured by other epidemiological sources. The people who make use of syringe exchange and other harm reduction services provide an important source of real-time information on the drugs currently being used, how they are being used, the effects of the current supply, and information on new drugs and drug use patterns. For example, xylazine was first reported by SSP service recipients in Burlington, Vermont at least twelve months before it was reported by treatment providers. Data from SSP service recipients is a valuable source of information that can provide health care providers, community providers, and health departments with an early warning system about emerging drug problems.

This study has several limitations that should be considered when interpreting these findings. One limitation is this study was conducted in one harm reduction program in a very small Northeastern city. As such, these findings should be considered in their environmental (e.g., geographic, sociodemographic) context and may not be generalizable to other regions, both within and outside of the state of Vermont. Compared to the Burlington metropolitan area with approximately 225,000 residents, much of the rest of the state is rural with 64.9% of residents living in rural areas [30]. Future research should be conducted to evaluate the generalizability of these findings to other areas of Vermont and the Northeast US.

A second limitation is that all data were collected via participant self-report which could have impacted the results in several different ways. Because drug use was based on self-report, its correspondence with objective measures is unknown and it is possible that participants may have used or not used other drugs (e.g., xylazine) unknowingly. Additionally, findings on current and past-year MOUD treatment should be interpreted while also considering that access to low-barrier MOUD is offered in the same building as this SSP. Finally, findings on routes of administration may have been impacted by the study population (i.e., SSP service recipients) and the availability of safer smoking supplies as another service [31].

## Conclusions

This study expands what is known about important sociodemographic, substance use, and health-related variables among individuals participating in a SSP in Vermont that also provides other harm reduction services. Specifically, these findings add to the literature by assessing differences in the types of drugs used and treatment participation—including MOUD—based on one’s primary (i.e., preferred) drug. Findings among this sample of service recipients indicate that polysubstance substance use was common [32], an important risk factor for experiencing a drug overdose [33, 34] that also has direct implications for the types of evidence-based interventions and services offered in this setting. Taken together, these findings highlight the important role of SSPs in providing community-based services [35] and provide knowledge that can inform future intervention services and treatment provision in this setting to help address the ongoing overdose epidemic.

### Supplementary Information

**Additional file 1**.** Table S1**. Drug Use Among Participants Currently in MOUD Treatment.

## Data Availability

The datasets generated and/or analyzed during the current study are not publicly available due to institutionally approved protocols but are available from the corresponding author on reasonable request.

## Abbreviations

US: United States
SSP: Syringe Service Program
HIV: Human Immunodeficiency Virus
HCV: Hepatitis C Virus
PWUD: People who use drugs
PWID: People who inject drugs
MOUD: Medication for opioid use disorder
