# Supplementary material for: Substance use patterns, sociodemographics, and health profiles of harm reduction service recipients in Burlington, Vermont
Source: Harm Reduct J. 2024 Apr 5;21:76. doi: 10.1186/s12954-024-00995-y (PMC10998322; doi:10.1186/s12954-024-00995-y)
Supplement: Supplementary file 1 — Additional file 1. Table S1. Drug Use Among Participants Currently in MOUD Treatment. [file 12954_2024_995_MOESM1_ESM.docx]

Supplemental Table 1. Drug Use Among Participants Currently in MOUD Treatment

| Substance | Current MOUD Treatment  (n = 87) | |
| --- | --- | --- |
|  | *N* | % |
| **Crack Cocaine** |  |  |
| *Used* | 77 | 88.5% |
| Injected | 7 | 9.1% |
| Smoked | 76 | 98.7% |
| Snorted | 1 | 1.3% |
| Not used | 10 | 11.5% |
| # Days used past week | 3.8 |  |
| # Days injected past week | 2.83 |  |
| **Powder Cocaine** |  |  |
| *Used* | 31 | 35.6% |
| Injected | 16 | 51.6% |
| Smoked | 5 | 16.1% |
| Snorted | 13 | 41.9% |
| Not used | 56 | 64.4% |
| # Days used past week | 1.24 |  |
| # Days injected past week | 1.63 |  |
| **Methamphetamine** |  |  |
| *Used* | 36 | 41.4% |
| Injected | 22 | 61.1% |
| Smoked | 20 | 55.6% |
| Snorted | 8 | 22.2% |
| Not used | 51 | 58.6% |
| # Days used past week | 3.63 |  |
| # Days injected past week | 3.41 |  |
| **Heroin** |  |  |
| *Used* | 55 | 63.2% |
| Injected | 38 | 69.1% |
| Smoked | 17 | 30.9% |
| Snorted | 12 | 21.8% |
| Not used | 32 | 36.8% |
| # Days used past week | 4.32 |  |
| # Days injected past week | 4.53 |  |
| **Fentanyl (by itself or mixed)** |  |  |
| *Used* | 60 | 69.0% |
| Injected | 36 | 60.0% |
| Smoked | 20 | 33.3% |
| Snorted | 9 | 15.0% |
| Not used | 27 | 31.0% |
| *Purposeful use?* |  |  |
| Yes | 20 | 23.0% |
| # Days used past week | 4.20 |  |
| # Days injected past week | 4.73 |  |
| **Xylazine** |  |  |
| *Used* | 29 | 33.3% |
| Injected | 18 | 62.1% |
| Smoked | 6 | 20.7% |
| Snorted | 3 | 10.3% |
| Not used | 58 | 66.7% |
| # Days used past week | 4.28 |  |
| # Days injected past week | 5.1 |  |
| **Alcohol** |  |  |
| *Used* | 41 | 47.1% |
| Not used | 46 | 52.9% |
| # Days used past week | 3.05 |  |
